# Inhibition of Interleukin-6 Receptor in a Murine Model of Myocardial Ischemia-Reperfusion

**DOI:** 10.1371/journal.pone.0167195

**Published:** 2016-12-09

**Authors:** Minke H. T. Hartman, Inge Vreeswijk-Baudoin, Hilde E. Groot, Kees W. A. van de Kolk, Rudolf A. de Boer, Irene Mateo Leach, Rozemarijn Vliegenthart, Herman H. W. Sillje, Pim van der Harst

**Affiliations:** 1 University of Groningen, University Medical Center Groningen, the department of Cardiology, Groningen, the Netherlands; 2 University of Groningen, University Medical Center Groningen, the Central Animal Facility, Groningen, the Netherlands; 3 University of Groningen, University Medical Center Groningen, the department of Radiology, Groningen, the Netherlands; Universidad de Buenos Aires, ARGENTINA

## Abstract

**Background:**

Interleukin-6 (IL-6) levels are upregulated in myocardial infarction. Recent data suggest a causal role of the IL-6 receptor (IL-6R) in coronary heart disease. We evaluated if IL-6R blockade by a monoclonal antibody (MR16-1) prevents the heart from adverse left ventricular remodeling in a mouse model of ischemia-reperfusion (I/R).

**Methods:**

CJ57/BL6 mice underwent I/R injury (left coronary artery ligation for 45 minutes) or sham surgery, and thereafter received MR16-1 (2mg/mouse) 5 minutes before reperfusion and 0.5mg/mouse weekly during four weeks, or control IgG treatment. Cardiac Magnetic Resonance Imaging (CMR) and hemodynamic measurements were performed to determine cardiac function after four weeks.

**Results:**

I/R caused left ventricular dilatation and a decrease in left ventricular ejection fraction (LVEF). However, LVEF was significantly lower in the MR16-1 treatment group compared to the IgG group (28±4% vs. 35±6%, p = 0.02; sham 45±6% vs. 43±4%, respectively; p = NS). Cardiac relaxation (assessed by dP/dT) was not significantly different between the MR16-1 and IgG groups. Also, no differences were observed in histological myocardial fibrosis, infarct size and myocyte hypertrophy between the groups.

**Conclusion:**

Blockade of the IL-6R receptor by the monoclonal MR16-1 antibody for four weeks started directly after I/R injury did not prevent the process of cardiac remodeling in mice, but rather associated with a deterioration in the process of adverse cardiac remodeling.

## Introduction

Myocardial infarction (MI) results in adverse cardiac remodeling and heart failure. Inflammation is an important process involved in the process of cardiac remodeling and might provide a target of therapy[1–4]. Currently, no pharmacotherapy specifically targeting inflammation in MI is available[5]. The cytokine interleukin-6 (IL-6) has both pro- and anti-inflammatory properties in inflammatory signaling pathways of various diseases[6]. IL-6 can activate intracellular signaling cascades such as the JAK/STAT and PI3K pathways in neutrophils and monocytes by binding to the IL-6R receptor. The PI3K pathway is associated with adaptive hypertrophy and protects cardiomyocytes from apoptosis[7,8]. Membrane-bound IL-6R is present in hepatocytes and leukocytes. The soluble IL-6 receptor (sIL-6R) has been found in human serum and forms a complex with IL-6 that can activate various cells lacking the membrane bound IL-6, a process called transsignaling[9]. IL-6 transsignaling is associated with pro-inflammatory roles of IL-6[10]. sIL-6R and IL-6 levels are associated with both cardiac injury and acute MI[11]. In acute coronary syndrome, high IL-6 levels were associated with reduced left ventricular ejection fraction[12]. In ST-segment elevation MI, IL-6 levels were higher in patients experiencing cardiovascular complications. Further, the sIL-6R gradient between the aorta and the coronary sinus was increased suggesting that sIL-6 is bound by the infarcted heart and affects the signal transduction of IL-6[13]. Recent genetic analysis using the Mendelian Randomization principle suggested that IL-6R signaling indeed plays a causal role in the development of coronary artery disease, suggesting IL-6R inhibition could be a potential new target in coronary artery disease[14,15]. In mice, the antibody MR16-1 has been found to inhibit IL-6R[16]. We hypothesize that blocking the IL-6R signaling pathway by MR16-1 in an experimental mouse model of myocardial ischemia-reperfusion (I/R) injury prevents adverse cardiac remodeling and preserves cardiac function.

## Methods

### Animals

All animal procedures were performed in accordance with and approved by the Committee of Animal Experimentation of the University of Groningen. In total 36 male C57Bl6/J mice were obtained from Harlan (Horst, the Netherlands) at an age of eight weeks, and randomly divided into four groups (N = 2x11 in ischemia groups, N = 2x7 in sham groups). During the entire experiment animals had ad libitum access to normal chow and water.

### Experimental protocol

Mice were anesthetized using Isoflurane gas (2.5%) and oxygen, and held on a heating mat of 37°C. The mice were mechanically ventilated (Harvard Minivent 845, vol: 250μl, freq: 180) after intubation. The heart was accessed via left thoracotomy and the pericardium was removed. Ischemia was induced by ligation of the left anterior descending coronary artery (LAD) with a 6–0 silk suture, tied onto a small piece of polyethylene-10 tubing to protect the myocardium for permanent ischemia after reperfusion. The suture was released after 60 minutes to allow reperfusion, and the muscle layer was closed using 5/0 prolene and the skinlayer was closed using 5/0 safil. After recovery of anaesthesia the mice received a single dose of the analgesic agent Buprenorphine (10μg/kg body weight) (Schering-Plough). Sham surgeries were identical except for the ligation of the LAD. Five minutes before the end of ischemic time, mice received an intravenous injection of 2 mg anti-mouse IL-6 receptor antibody MR16-1 (Chugai Pharmaceutical Co., Ltd.) or 2 mg control IgG (Jackson Immunoresearch, 012-000-003). Afterwards they were injected intraperitoneally once a week with a total of four injections with either 0.5 mg MR16-1 or 0.5 mg IgG. The specificity and blocking ability of this monoclonal antibody were confirmed in previous reports[16,17]. After four weeks, cardiac structure and function was evaluated by Cardiac Magnetic Resonance imaging (CMR) (9.4-Tesla, 89-mm diameter magnet, 1500mT/m gradient, 400 MR system Bruker Biospin, Ettlingen, Germany).

### Cardiac Magnetic Resonance imaging

Mice were anesthetized with 1.5–3 vol% Isoflurane in a 2:1 mixture of air (0.5l/min) and oxygen (0.25l/min) during CMR performed within 28 days after surgery. Mice were imaged in a vertical 9.4-Tesla, 89-mm diameter bore size scanner equipped with a 1500 mT/m gradientset and connected to an advanced 400 MR system (Bruker BioSpin, Ettlingen, Germany) using a quadrature-driven birdcage coil with an inner diameter of 3cm. Cardiac and respiration motion signals were derived from a pressure pad placed under the chest of the mouse and monitored by an electrocardiography (ECG) Trigger Unit (RAPID biomedical GmBH, Germany). Heart rate was maintained between 400–600 beats per minute and respiration rate between 20–60 breaths per minute. ParaVision 4.0 and IntraGate software (Bruker BioSpin GmBH, Germany) were used for cine MR acquisition and reconstruction. In the end, short-axis (oriented perpendicular to the septum) cardiac cine MR images were acquired. To cover the entire heart from apex to base, 7 slices (sham) or 7–9 slices (ischemia-reperfusion) were needed, with slice thickness of 1mm without gaps. Cardiac function analyses were performed using QMass 7.6 software (© 2014 Medis Medical Imaging Systems B.V., Leiden, the Netherlands). All analyses were performed by an experienced observer, blinded for treatment, and reviewed by a radiologist. Papillary muscles were included from the blood pool with the manual method, which decreases intra- and inter observer variability in left ventricular (LV) volume and mass[18]. Cardiac function parameters included left ventricular end diastolic volume (LVEDV), left ventricular end systolic volume (LVESV), left ventricular ejection fraction (LVEF), left ventricular mass (LVM) and stroke volume.

### Hemodynamic measurements

Prior to sacrifice, hemodynamic measurements were performed using a PV-loop catheter (SciSense, 1.2F). Hemodynamic measurements were analysed using LabChart 7 software (version 7.2, ADinstruments, New Zealand) according to a standardized protocol described previously[19]. Pressure in the aorta and left ventricle was calculated for all animals. Within a week after CMR measurements blood was sampled via heart puncture. After heart puncture, the abdomen was opened and the mouse was perfused with 0.9% NaCl via the apex. Subsequently, hearts and tibia were harvested and weighed. Tissues were snap frozen in liquid nitrogen for molecular assays or formalin fixed for immunohistochemistry.

### Histology

For immunohistochemical analysis, midslices of the left ventricle were fixed in 4% paraformaldehyde for 24 hours as described[20]. After fixation and embedding into paraffin, 4 μm thin slices were cut and stained with Masson trichrome staining for the analysis of collagen deposition. The whole left ventricle section was imaged for quantification using a microscope (Nanozoomer 2.0-HT, Hamamastu, Japan) and subsequently the Aperio’s ImageScope software (ScanScope, Aperio Technologies, Vista, CA, USA) was used for determining the fibrotic area. The percentage of the total left ventricle with fibrosis was determined. To evaluate cardiomyocytes hypertrophy, cell membrane staining with wheat germ agglutinin–FITC (WGA-FITC) was performed and cross sectional cardiomyocyte diameter was determined using an immunofluorescence microscope (Leica, DM 4000B, Germany). An average of 50 cells were evaluated per section. The infarct size was determined as the ratio between the fibrotic area (μm^2^) and total LV area (μm^2^).

### Plasma IL-6 determination

To determine plasma IL-6 levels, 50 μL EDTA-plasma samples from blood drawn directly after sacrifice of the mice were used. A mouse IL-6 Quantikine ELISA kit was used according to the instructions of the manufacturer (R&D systems, Minneapolis, USA).

### Statistical analysis

Variables are presented with mean and standard deviation when distribution was normal. When normal distribution could not be assumed, variables are presented with median and interquartile range (IQR). Statistical analyses were performed using the one-way Analysis of Variance with the post hoc Bonferroni or the Kruskal-Wallis test following the Dunn’s test for multiple comparisons under Bonferroni, when appropriate. All reported *P* values were 2-sided and *P*<0.05 was considered to indicate a significant difference between groups. When not reported in the text, *P* values are listed in the S1 Table. Analyses were performed using STATA/IC version 13.0 (StataCorp LP, College Station, Texas, USA).

## Results

### General characteristics

In total 36 mice were included in the experiment and underwent surgery. Four mice died during surgery before receiving any treatment. No mice died between first treatment and termination. One mouse in the I/R-IgG group with worse LVEF and more severe left ventricular (LV) dilatation than others, as seen in permanent infarction was excluded from further analysis during blinded evaluation of the CMR data.

### CMR and hemodynamic measurements

#### Ischemia-reperfusion vs sham

Parameters of cardiac function including LVEF, LVEDV, LVESV, LVM, the rate of LV pressure rise and fall (LV dP/dT min, LV dP/dT max) and LV end diastolic pressures were significantly impaired in the I/R groups compared to the sham groups (Table 1). When not reported in the text, P values are listed in the S1 Table ‘P-values’. Stroke volume, maximum pressure in the aorta were also lower in both I/R compared to both sham groups (IgG groups p = 0.01, MR16-1 groups p = 0.04).

**Table 1:** Cardiac function, LV and aortic pressures 28 days after surgery.

Parameter	Sham IgG	I/R-IgG	Sham MR16-1	I/R-MR16-1
Mean ± SD/ IQR (25;75)				
***CMR cardiac function***	*n = 7*	*n = 8*	*n = 7*	*n = 9*
**LVEF (%)**	43 ± 4	35 ± 6**†**	45 ± 6	28 ± 4‡*
**LVEDV (μl)**	59 ± 6	75 ± 16**†**	60 ± 5	78 ± 10**‡**
**LVESV (μl)**	32 (31;35)	50 (42;61)**†**	33 (28;41)	56 (54;60)**‡**
**Stroke volume (μl)**	25 ± 3	26 ± 5	27 ± 2	21 ± 3**‡**
**LVM (mg)**	78 (75;82)	89 (84;104)**†**	77 (76;81)	91 (85;93)**‡**
***Hemodynamic measures***	*n = 5–7*	*n = 7–8*	*n = 7*	*n = 9*
**dP/dT max (mmHg/s)**	8425 (8227;9099)	6506 (6065;6736)**†**	8940 (7771;9180)	5797 (5440;6522)**‡**
**dP/dT min (mmHg/s)**	-7921 ± 1273	-6079 ± 766**†**	-7684 ± 1245	-5102 ± 766**‡**
**dP/dT max index (1/s)**	80 ± 6	63 ± 7**†**	81 ± 8	62 ± 5**‡**
**dP/dT min index (-1/s)**	-81 ± 14	-59 ± 6**†**	-80 ± 12	-53 ± 6**‡**
**Tau**	6.9 ± 0.8	9.6 ± 1.1**†**	6.6 ± 1.5	11.0 ± 1.6**‡**
**LV EDP (mmHg)**	9 ± 2	13 ± 2**†**	8 ± 2	13 ± 3**‡**
**LV ESP (mmHg)**	101 (97;101)	100 (97;101)	98 (92;100)	95 (92;99)
**Pmax aorta (mmHg)**	109 ± 6	101 ± 4	106 ± 5	100 ± 7**‡**
**Pmin aorta (mmHg)**	75 ± 5	75 ± 3	73 ± 4	74 ± 6

**†** = I/R-IgG vs. sham p<0.05

**‡** = I/R-MR16-1 vs. sham p<0.05

* = I/R-MR16-1 vs. I/R-IgG p<0.05.

SD = standard deviation; I/R = ischemia reperfusion; LVEF = left ventricular ejection fraction; LVEDV = left ventricular end diastolic volume; LVESV = left ventricular end systolic volume; LVM = left ventricular mass; dP/dT = delta pressure / delta time; LV EDP = left ventricular end diastolic pressure; LV ESP = left ventricular end systolic pressure; Pmax = maximum pressure; Pmin = minimum pressure.

#### MR16-1 vs IgG ischemia-reperfusion

**Fig 1:**
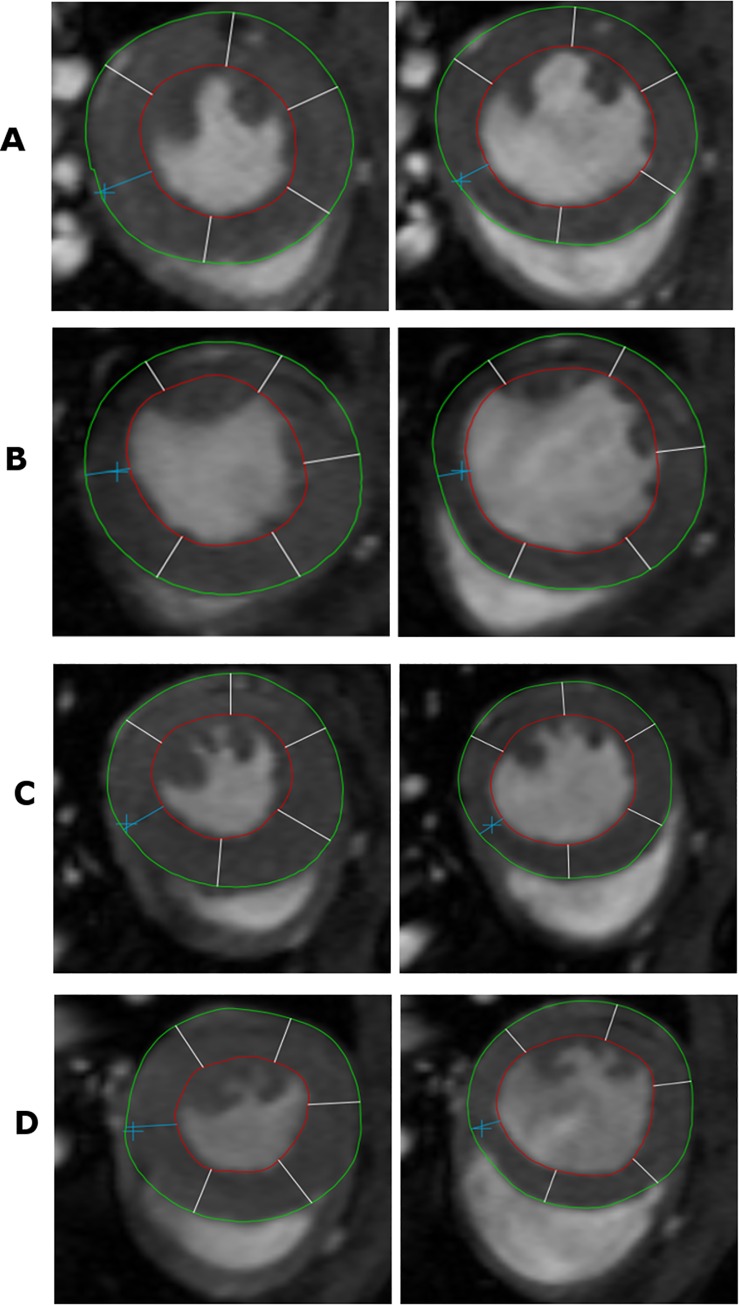
End diastolic and end systolic contours of the left ventricle. Effect of I/R in the MR16-1 and control IgG groups on cardiac function as measured by Cardiac Magnetic Resonance Imaging and visualized with mean and standard deviation; (A) Average case of the control IgG I/R group with end systolic and end diastolic contours, volumes and LVEF (LVESV 57 μl, LVEDV 85 μl, LVEF 32%); (B) Average case of the MR16-1 treatment I/R group with end systolic and end diastolic contours, volumes and LVEF (LVESV 60 μl, LVEDV 81 μl, LVEF 26%); (C) Average case of the IgG sham group with end systolic and end diastolic contours, volumes and LVEF (LVESV 32 μl, LVEDV 56 μl, LVEF 43%); (D) Average case of the MR16-1 sham group with end systolic and end diastolic contours, volumes and LVEF (LVESV 33 μl, LVEDV 60 μl, LVEF 44%).

LVEF in the MR16-1 I/R group was significantly more reduced than in the IgG-I/R group (mean LVEF of 28±4% and 35±6%, respectively, p = 0.02) (Table 1 & Fig 1). LV dilatation as indicated by LVEDV and LVESV did not differ between the I/R-IgG and I/R-MR16-1 groups. In addition, no differences were observed between I/R groups in remaining LV systolic function parameters, including dP/dT max and LV diastolic function parameters, including LV end diastolic pressure and Tau. LV end-systolic pressures and aortic pressures were also not different between the I/R-IgG and I/R-MR16-1 groups.

### Organ weights and histology

**Fig 2:**
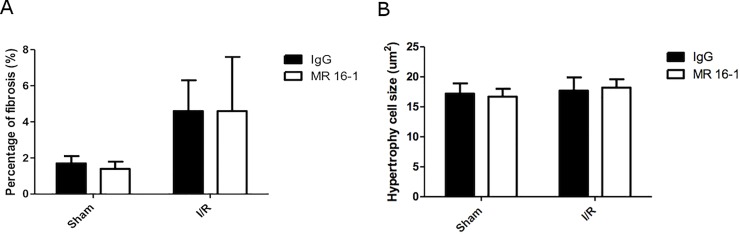
Fibrosis and hypertrophy. Effect of I/R vs. sham in the MR16-1 and IgG groups on percentage of fibrosis and cell size and visualized with mean and standard deviation; I/R resulted in an increase in the percentage of fibrosis, however there was no effect of MR16-1 vs. IgG among these groups (A); No effect on cell size was observed in all groups (B).

Within the two sham surgery groups, heart weights, corrected for tibia length, were similar *(Table 2)*. Mice in the I/R-MR16-1 group had a lower body weight compared to mice in the sham group (p = 0.003). Furthermore, LV weight corrected for tibia length was increased in the ischemia groups compared to mice in the sham groups (p≤0.03). There were no significant differences in organ weights between the I/R-MR16-1 and I/R-IgG groups. Masson staining and myocyte cell size measurements were performed to assess the area of infarct, severity of myocardial fibrosis and cellular hypertrophy. Infarct size did not differ between the I/R-IgG and I/R-MR16-1 groups. The percentage of fibrosis was higher in the I/R groups compared to the sham groups (approximately 2-fold increase) although myocyte cell size did not significantly change *(Table 2 & Fig 2)*. No d**i**fferences in either fibrosis or cell size were observed between the I/R-IgG and I/R-MR16-1 groups.

**Table 2:** Organ weights and histology 28 days after surgery.

Parameter	Sham IgG	I/R IgG	Sham MR16-1	I/R MR16-1
Mean ± SD/ IQR (25;75)				
***Organ weights***	*n = 7*	*n = 8*	*n = 7*	*n = 9*
**Body weight (g)**	28.4 ± 1.0	27.1 ± 1.0	28.0 ± 1.3	26.6 ± 1.0‡
**Tibia length (mm)**	18.2 ± 0.3	18.2 ± 0.5	18.5 ± 0.2	18.1 ± 0.4
**Atria W/TL (mg/mm)**	0.4 (0.4;0.5)	0.5 (0.5;0.6)**†**	0.4 (0.4;0.5)	0.5 (0.4;0.6)
**Right ventricle W/TL (mg/mm)**	1.4 ± 0.1	1.5 ± 0.3	1.3 ± 0.1	1.6 ± 0.2
**Left ventricle W/TL (mg/mm)**	6.2 (6.0;6.7)	7.1 (6.9;7.9)**†**	6.0 (5.6;6.2)	6.6 (6.4;6.7)‡
***Histology***	*n = 6–7*	*n = 8*	*n = 6–7*	*n = 8*
**Infarct size (per μm^2^)**	0.01 ± 0.01	0.06 ± 0.05**†**	0 ± 0	0.07 ± 0.05**‡**
**Fibrosis (%)**	1.7 ± 0.4	4.6 ± 1.7**†**	1.4 ± 0.4	4.6 ± 3.0**‡**
**Cell size (μm^2^)**	17.2 ± 1.7	17.7 ± 2.2	16.7 ± 1.3	18.2 ± 1.4

† = I/R-IgG vs. sham p<0.05

‡ = I/R-MR16-1 vs. sham p<0.05

I/R = ischemia reperfusion, W = weight, TL = tibia length.

### IL-6 plasma levels

Previous IL-6R studies showed elevation of plasma IL-6 levels in response to IL-6R receptor blockage [21–23]. To confirm the antibody blocking ability of MR16-1 at the doses used in this study plasma IL-6 levels were determined. As shown in S1 Fig ‘IL-6 plasma levels in MR16-1 and IgG treated animals’, IL-6 levels were significantly higher in MR16-1 treated animals (1.1 ± 1.2 pg/mL in sham IgG vs. 8.8 ± 4.1 pg/mL in sham MR16-1 group, p = 0.001; and 2.2 ± 1.1 pg/mL in I/R IgG vs. 12.0 ± 3.6 pg/mL in I/R M16-1 group, p<0.001).

## Discussion

This is the first experimental study evaluating the effect of interleukin-6 receptor inhibition on left ventricular remodeling in a myocardial I/R model. Inhibition of IL-6R with MR16-1 treatment after I/R resulted in reduced systolic LV function. No differences in myocardial fibrosis, infarct size and hypertrophy were observed between these two treatment groups. Our data show that IL-6R blocking, initiated directly after MI and continued for four weeks, does not result in preserved cardiac function in this experimental myocardial I/R model. Our data contradict previous experimental data obtained in a murine model.

Differences in pathogenesis related to the chosen experimental model or dosage and treatment regime could have caused the discrepancies in outcome. In a previous study, mice were injected intraperitoneally with MR16-1 or control IgG 0.5mg/body after permanent coronary artery ligation[24]. In this study, treatment with IL-6R antibody was associated with improved fractional shortening and smaller LV end diastolic diameter, as assessed with echocardiography. Furthermore, the treatment group had a higher survival rate after four weeks compared to the control group (resp. 80.6% vs. 59.5%). We aimed at extending these findings to a more clinically relevant model, of I/R, which is more comparable to patients presenting with a MI and subsequently treated with percutaneous coronary intervention[5].

Apart from differences in the study design and used mouse strain, the lack of preservation of LV function after MR16-1 treatment in our model may be related to the evaluation of LV function with CMR and invasive hemodynamic measurements, instead of assessment by echocardiography and by differences in timing of treatment (directly after induction of ischemia vs. five minutes before reperfusion) treatment dose (0.5 mg/body vs. 2.0 mg/body at baseline), treatment duration (single injection vs. four-week long). MR16-1 treatment was associated with increased plasma IL-6 levels, a result of the delayed clearance from the blood after IL-6R blockade with MR16-1[21–23], indicating that our treatment regimen worked properly. Nevertheless, prolonged timing of treatment could have influenced the outcome. Increase of inflammatory monocytes after MI has been associated with cardiac remodeling. Monocytes activated during the first four days after MI are said to be inflammatory, yet between day four and day eight after MI reparative monocytes triggering collagen deposition are increasingly present[25]. In one study in ST-segment elevation MI (STEMI) patients evaluating early and late initiation of statin therapy, late initiation was associated with less anti-inflammatory effects, suggesting inflammation targeted therapy started early after MI is favorable[26]. In our study treatment was continued until four weeks after MI, thus inhibiting potential beneficial processes initiated by reparative monocytes in the later phase after MI. In our study we did not observe an effect on infarct size measured by area of fibrosis after 4 weeks. However, our study is limited by the fact that we did not include additional groups sacrificed on day 1 after coronary artery ligation to evaluate area at risk and infarct size. Finally, MR16-1 does not selectively block IL-6 transsignaling involving pro-inflammatory sIL-6R, but supposedly also blocks IL-6 classic signaling via membrane bound anti-inflammatory IL-6R[16,27]. The importance of IL-6 transsignaling is illustrated by an experimental model where IL-6 transsignaling was selectively blocked with the fusion protein sgp130Fc and resulted in regression of atherosclerosis[28]. Sgp130 is thought to be the natural inhibitor of sIL-6R[10]. Moreover, in a heart failure cohort soluble glycoprotein 130 (sgp130) levels were associated with mortality, whereas plasma IL-6 was not[29]. Recently, IL-6 transsignaling inhibition by sgp130 was put forward as a potential new treatment in heart failure[30]. On the other hand, in an I/R study, rats underwent IL-6, s-IL-6R, IL-6/sIL-6R complex suppletion or control pretreatment prior to coronary artery occlusion resulted in reduced infarct size only in the IL-6/sIL-6R complex group[31]. This alternative approach of IL-6R complex suppletion for reduction of infarct size appears to be in accordance with our findings that inhibition of IL-6R is potentially harmful for cardiac function. However, some authors have suggested that increased IL-6/sIL-6R leads to an increase in sgp130 levels thus controlling IL-6 transsignaling[30]. In humans, the IL-6R can be blocked with Tocilizumab, a FDA approved drug for the treatment of several inflammatory diseases, including rheumatoid arthritis[32]. Recently, a trial has been published evaluating the effect of a single gift of Tocilizumab on high sensitivity C-reactive protein (hs-CRP) area under the curve in patients with non-STEMI[33]. The levels of hs-CRP were indeed more than twice as high in the placebo compared to the treatment group. This suggests the inflammatory response in non-STEMI can be attenuated by Tocilizumab. In spite of that, it did not translate into an improvement of LVEF or LV dimensions after 6 months of follow-up. Future clinical trials studying the effect of Tocilizumab in MI should consider to opt for cardiac function as primary endpoint. This could answer the question whether Tocilizumab is able to prevent adverse cardiac remodeling by attenuating the inflammatory response. Apart from that, further studies should evaluate whether preservation of cardiac function after MI might be achieved only by focusing on selective IL-6 transsignaling inhibition targets.

## Conclusion

IL-6R inhibition by MR-16 antibody treatment for four weeks after I/R reduced LV function in an experimental mouse model. This suggests that acute, continuous blockade of the IL-6R pathway might have a potentially negative effect on cardiac remodeling after MI. Our study cannot exclude a potential beneficial effect of IL-6R inhibition when administered in the early period after MI. In addition, the role of IL-6R inhibition in the development and progression of atherosclerosis cannot be deducted from our data.

## Supporting Information

S1 Fig (DOCX)

S1 Table (DOCX)
