# Supplementary material for: Inhibition of Interleukin-6 Receptor in a Murine Model of Myocardial Ischemia-Reperfusion
Source: PLoS One. 2016 Dec 9;11(12):e0167195. doi: 10.1371/journal.pone.0167195 (PMC5147868; doi:10.1371/journal.pone.0167195)
Supplement: S1 Fig — (DOCX) [file pone.0167195.s001.docx]

### Supplementary Figure 1. IL-6 plasma levels in MR16-1 and IgG treated animals

Figure legend

Plasma levels of IL-6 were significantly higher in MR16-1 treated animals as visualized with mean values and concomitant standard errors (1.1 ± 1.2 pg/mL in sham IgG (*n*=6) vs. 8.8 ± 4.1 pg/mL in sham MR16-1 group (*n*=6), p=0.001; and 2.2 ± 1.1 pg/mL in I/R IgG (*n*=7) vs. 12.0 ± 3.6 pg/mL in I/R M16-1 group (*n*=8), p<0.001).
