# Supplementary material for: Inhibition of Interleukin-6 Receptor in a Murine Model of Myocardial Ischemia-Reperfusion
Source: PLoS One. 2016 Dec 9;11(12):e0167195. doi: 10.1371/journal.pone.0167195 (PMC5147868; doi:10.1371/journal.pone.0167195)
Supplement: S1 Table — (DOCX) [file pone.0167195.s002.docx]

### Supplementary Table 1. P-values

| Parameter | MR16-1 sham vs. IgG sham (*p*) | I/R-IgG vs. sham  (*p*) | I/R-MR16-1 vs. sham (*p*) | I/R-MR16-1 vs.  I/R-IgG (*p*) |
| --- | --- | --- | --- | --- |
| LVEF (%) | 1.000 | 0.001 | <0.001 | 0.020 |
| LVEDV (µl) | 1.000 | 0.007 | 0.001 | 1.00 |
| LVESV (µl) | 1.000 | 0.018 | <0.001 | 0.389 |
| Stroke volume (µl) | 1.000 | 1.000 | 0.012 | 0.062 |
| LV mass (mg) | 1.000 | 0.007 | 0.005 | 1.000 |
| dP/dT max | 1.000 | 0.005 | <0.001 | 0.634 |
| dP/dT min | 1.000 | 0.003 | <0.001 | 0.196 |
| dP/dT max index | 1.000 | <0.001 | <0.001 | 1.000 |
| dP/dT min index | 1.000 | <0.001 | <0.001 | 0.527 |
| Tau | 1.000 | <0.001 | <0.001 | 0.131 |
| LV EDP | 0.990 | <0.001 | <0.001 | 1.000 |
| LV ESP | 0.337 | 0.992 | 0.639 | 0.400 |
| Pmax aorta | 0.734 | 0.066 | 0.006 | 1.000 |
| Pmin aorta | 0.940 | 1.000 | 1.000 | 1.000 |
| Body weight | 1.000 | 0.080 | 0.003 | 0.859 |
| Tibia length | 0.581 | 1.000 | 1.000 | 0.439 |
| Atria/LT | 0.976 | 0.023 | 0.225 | 0.516 |
| RV/LT | 1.000 | 0.198 | 0.147 | 1.000 |
| LV/LT | 0.400 | <0.001 | 0.030 | 0.373 |
| Infarct size per µm^2^ | 1.000 | 0.008 | 0.002 | 1.000 |
| Fibrosis (%) | 1.000 | 0.002 | 0.003 | 1.000 |
| Cell size (µm^2^) | 1.000 | 0.940 | 0.330 | 1.000 |
